# Quercetin Supplementation Improves Intestinal Digestive and Absorptive Functions and Microbiota in Rats Fed Protein-Oxidized Soybean Meal: Transcriptomics and Microbiomics Insights

**DOI:** 10.3390/ani14162326

**Published:** 2024-08-12

**Authors:** Zhiyong Wang, Peng Wang, Yanmin Zhou, Su Zhuang

**Affiliations:** College of Animal Science and Technology, Nanjing Agricultural University, No. 1 Weigang, Nanjing 210095, China; 2021205034@stu.njau.edu.cn (Z.W.); 2020205032@stu.njau.edu.cn (P.W.); zhouym@njau.edu.cn (Y.Z.)

**Keywords:** quercetin, protein oxidation, soybean meal, microbiota

## Abstract

**Simple Summary:**

The protein oxidation of soybean meal, resulting from storage, can detrimentally impact the intestinal digestive and absorptive functions in animals. Meanwhile, quercetin can regulate protein and lipid metabolism, intestinal transporter numbers, and gut microbiota in animals. The aim of this study was to investigate the effect of quercetin supplementation on the intestinal digestive and absorptive functions and microbiota in rats fed protein-oxidized soybean meal. The results of this study indicated that the protein-oxidized soybean meal decreased the relative weights of the digestive organs and the duodenal villus height, thereby reducing the apparent ileal digestibility of amino acids in the rats. Transcriptomics and microbiomics revealed that quercetin supplementation alleviated these adverse effects primarily by upregulating the pathway of intestinal amino acid transmembrane transporter activity and improving the cecal microbial composition.

**Abstract:**

To clarify the nutritional mechanisms of quercetin mitigation in the digestive and absorptive functions in rats fed protein-oxidized soybean meal, 48 three-week-old male SD rats were randomly allocated into a 2 × 2 factorial design with two soybean meal types (fresh soybean meal or protein-oxidized soybean meal) and two quercetin levels (0 or 400 mg/kg) for a 28-day feeding trial. The protein-oxidized soybean meal treatment decreased (*p* < 0.05) the relative weights of the pancreas, stomach, and cecum, duodenal villus height, pancreatic and jejunal lipase activities, apparent ileal digestibility of amino acids, and apparent total tract digestibility of dry matter, crude protein, and ether extract. The supplementation of quercetin in the protein-oxidized soybean meal diet reversed (*p* < 0.05) the decreases in the duodenal length, ileal villus height, lipase activity, apparent ileal digestibility of amino acids, and apparent total tract digestibility of dry matter, crude protein, and ether extract. Transcriptomics revealed that the “alanine transport” and “lipid digestion and absorption” pathways were downregulated by the protein-oxidized soybean meal compared with fresh soybean meal, while the “basic amino acid transmembrane transporter activity” and “lipid digestion and absorption” pathways were upregulated by the quercetin supplementation. Microbiomics revealed that the protein-oxidized soybean meal increased the protein-degrading and inflammation-triggering bacteria in the cecum, while the relative abundances of beneficial bacteria were elevated by the quercetin supplementation.

## 1. Introduction

Soybean meal (S) is a predominant source of protein in animal feed. However, the protein of S is subject to oxidation reactions during production, processing, and storage [1,2], and its nutritional value is also affected by the extent of the oxidation. Protein oxidation is a complex series of reactions that can lead to alterations in the molecular structure of proteins [1], ultimately affecting their amino acid availability [3] and overall nutritional value [4,5]. Previous research has shown that heat-induced protein-oxidized soy protein isolate (SPI) could lead to a decrease in body weight gain, relative jejunum weight, and pancreatic trypsin activity [6]. Other researchers found that heat-induced protein-oxidized soybean meal (OS) decreased the average daily gain (ADG) and apparent total tract digestibility (ATTD) of crude protein (CP) and dry matter (DM) and increased the feed conversion ratio (F:G) in broilers [7]. Additionally, studies have reported that heat-induced OS decreased the mRNA expression levels of amino acid transporters, glucose transporters, and peptide transporters in the intestinal mucosa of laying hens [8].

To improve these adverse effects in animals fed OS, we performed screening experiments in vitro to determine whether quercetin (Q) could be tried as a feed additive in the OS diet. Q is a polyphenolic compound extracted from plants that has a variety of biological functions, mainly including antioxidant and immune functions [9,10]. Moreover, Q has been reported to increase the productive performance in broilers [11] and laying hens [12]. Furthermore, Q has beneficial impacts on regulating protein and lipid metabolism [13,14], the mRNA expression of nutritional transporters [11], and the gut microbial composition [11,15]. Transcriptomics can obtain a large amount of biological information through high-throughput gene expression sequencing, such as genome annotation [16]. Microbiome analysis can be used to explore functional differences in the gut microbiota [17], and how diet affects the interspecies variation in the microbial composition [18].

The beneficial effects of Q on the animal performance, nutrient digestion and absorption, and gut microbial composition have been found; however, little is known about how the OS diet with the addition of Q influences the gene expression related to the jejunal digestive and absorptive functions and cecal microbial composition in rats. Thus, the aim of this study was to utilize omics technology to investigate the effect of Q on improving the intestinal digestive and absorptive functions and microbial composition in rats fed OS.

## 2. Materials and Methods

### 2.1. Materials and Chemicals

Fresh soybean meal (FS) was purchased from Yihai Cereal & Oil Industry Co., Ltd. (Lianyungang, China). The contents of moisture, crude protein (CP), ether extract (EE), and ash in the FS were 12.99%, 43.13%, 0.88%, and 6.04%, respectively. FS was randomly divided into two parts. One was stored in a −20 °C refrigerator to inhibit protein oxidation [19], while the other was stored in a constant temperature and humidity chamber at 35 °C and 60% humidity to induce protein oxidation. The chamber was disinfected with 75% alcohol wiping and UV lamp irradiation before the storage test. All samples were also disinfected with UV lamps prior to storage and after stirring every 7 days. After 56 days, the protein carbonyl values in the FS and OS were 7.09 nmol/mg protein and 10.32 nmol/mg protein, respectively [20]; the contents of moisture in the FS and OS were the same [21]; the contents of Aspergillus flavus B1 toxin in the FS and OS were both lower than the national limit standard (30 μg/kg); and the contents of other toxins were also lower than the national limit standard (Feed Hygiene Standard, GB 13078-2017) [22]. Q (95% purity) was sourced from Hunan E.K. Herb Co., Ltd. (Changsha, China).

### 2.2. Animals, Experimental Design, Diets, and Management

All experimental protocols were approved by Nanjing Agricultural University’s Animal Care and Use Committee (registered number, 20220920156). Due to the complexity and instability of the experiment will be increased by the physiological status of the female animals (e.g., estrous cycle, mating, pregnancy, lactation, etc.), only male animals were used in this experiment. 48 three-week-old male Sprague–Dawley rats weighing 55 ± 5 g, purchased from Jiangsu Wukong Biotechnology Co., Ltd. (Nanjing, China), were randomly divided into four groups with 12 replicates per group and one rat per replicate for 35 days, including a 7-day pre-test period and 28 d test period. The rats were fed 4 treatments diets (according to a 2 × 2 factorial design): FS, FS + Q, OS, and OS + Q. The ingredient compositions and nutrient contents of the basal diets are presented in Table 1. The quercetin-added contrast diets consisted of adding 400 mg quercetin premix per kilogram of the corresponding basal diets. An inert marker, Cr_2_O_3_ (0.3% feed, as-fed basis), was included in each tested diet to facilitate the calculation of the nutrient apparent digestibility. The diets were prepared by Jiangsu Xietong Pharmaceutical Bio-engineering Co., Ltd. (Nanjing, China). Animals were raised in cages at a specific pathogen-free (SPF) grade and under conditions of 20 ± 2 °C temperature, 50–70% humidity, and a 12 h light–dark cycle, with ad libitum access to food and water.

### 2.3. Sample Collection

On the 35th day, the fecal samples were collected and stored at −20 °C for analysis of the apparent total tract digestibility (ATTD) of crude protein (CP), dry matter (DM), and ether extract (EE). All rats were weighed, anesthetized with ether, and euthanized by cervical dislocation. Then, digestive glands (pancreas and liver) and digestive organs (stomach, duodenum, jejunum, ileum, cecum, and colorectum) were excised and weighed, and the lengths of the intestinal tissues were measured. A part of the samples (pancreas, intestinal tissues, ileal digesta, and cecal digesta), except for intestinal tissue segments for morphological analysis, were immediately frozen in liquid nitrogen and stored at −80 °C for further analysis.

### 2.4. Growth Performance and Relative Organ Weight Measurement

Feed intake was measured every two days to calculate the average daily feed intake (ADFI). Body weights were measured weekly to calculate the average daily gain (ADG) and feed conversion ratio (F:G). The ADFI, ADG, F:G, and relative organ weight formulae were calculated using the following equations:

ADG = (final body weight − initial body weight)/number of days of the rearing period;

ADFI = (feed offer weight − feed residue weight)/number of days of the rearing period;

F:G = ADFI/ADG;

Relative organ weight = organ weight/body weight.

### 2.5. Intestinal Morphological Analysis

The intestinal tissue segments were fixed in 4% paraformaldehyde and then embedded in paraffin, sliced, placed on glass slides, and stained with hematoxylin and eosin (H&E). Villi heights and crypt depths were assessed using a Nikon Eclipse E100 microscope (Nikon, Tokyo, Japan) and ScopeImage 9.0 image-processing software (Bioimager Inc., Richmond Hill, ON, Canada), with 12 replicates tested in each group.

### 2.6. Determination of Digestive Enzyme Activity

Pancreatic and jejunal tissues were separately homogenized with ice-cold normal saline solution in appropriate proportions. Homogenates of pancreatic and jejunal tissues were centrifuged according to the conditions described in the kit protocols, and the supernatants were utilized to evaluate trypsin, lipase, and α-amylase activities, respectively. All procedures were conducted in accordance with the protocols provided by the manufacturer (Nanjing Jiancheng Bioengineering Institute, Nanjing, China). The trypsin activity was tested with a trypsin assay kit (Cat. No.: A080-2-2); the lipase activity was tested with a lipase assay kit (Cat. No.: A054-1-1); the α-amylase activity was tested with an α-amylase assay kit (Cat. No.: C016-1-1). 

### 2.7. Determination of Nutrient Apparent Digestibility

According to the AOAC method, the contents of AAs (code 994.12) in the diets and ileal digesta, and the DM (code 2001.12), CP (code 2001.11), and EE (code 2003.06) in the diets and feces, were analyzed. The content of DM was determined by weight loss after 12 h in an oven at 105 °C; the content of CP was accurately measured using a Kjeldahl nitrogen determination instrument (Kjeltec8400, FOSS, Hilloeroed, Denmark); the content of EE was determined using a Soxhlet extraction device; the content of AA was analyzed using an amino acid analyzer (LA8080, HITACHI, Tokyo, Japan); the content of Cr_2_O_3_ was measured using an inductively coupled plasma spectrometer (ICAP7000, Thermo Scientific, Waltham, MA, USA).

AID_(N)_ or ATTD_(N)_ = 1 − (Cr_(D)_/Cr_(I or F)_) × (N_(I or F)_/N _(D)_)

where AID_(N)_ or ATTD_(N)_ is the AID or ATTD of nutrient; Cr_(D)_ is the content of Cr_2_O_3_ in the diets; Cr_(I or F)_ is the content of Cr_2_O_3_ in the ileal digesta or feces; N_(I or F)_ is the content of nutrient in the ileal digesta or feces; N_(D)_ is the content of nutrient in the diets [23].

### 2.8. Transcriptomics Analysis

Total RNA was extracted from the jejunal tissues using the Trizol Reagent (Invitrogen, Carlsbad, CA, USA, Cat. No.: 15596026CN). The concentration, quality, and integrity of the RNA were determined using a NanoDrop spectrophotometer (Thermo Fisher Scientific, Waltham, MA, USA) and agarose gel electrophoresis. An NEBNext Ultra II RNA Library Prep Kit for Illumina (New England Biolabs, Ipswich, MA, USA, Cat. No.: E7770S) was used to construct cDNA libraries, according to the manufacturer’s instructions. Products were purified using the AMPure XP system (Beckman Coulter, Beverly, CA, USA) and were quantified using the high-sensitivity DNA kit (Agilent, Palo Alto, CA, USA, Cat. No.: 5067-4626) on a Bioanalyzer 2100 system (Agilent, Palo Alto, CA, USA). The final sequencing library was sequenced on the NovaSeq 6000 platform (Illumina, San Diego, CA, USA) at Personal Bio Inc. (Shanghai, China). All sequence data have been submitted to the NCBI under BioProject ID PRJNA1121032.

### 2.9. Microbiomics Analysis

Total genomic DNA was extracted from the cecal digesta samples using the Stool DNA Kit (Omega Bio-Tek, Norcross, GA, USA, Cat. No.: D4015-02), according to the manufacturer’s protocols. The quantity and quality of the extracted DNAs were determined using the same method as that of the transcriptomics analysis. The bacterial 16S rRNA gene V3–V4 region was amplified via polymerase chain reaction. The forward primer 338F and the reverse primer 806R were used for this amplification. The amplified products were purified using VAHTSTM DNA Clean Beads (Vazyme, Nanjing, China). The purified products were then quantified employing the Quant-iT PicoGreen dsDNA Assay Kit (Invitrogen, Carlsbad, CA, USA, Cat. No.: P7589). Sequencing was conducted at Personal Bio Inc. (Shanghai, China) using the Illlumina NovaSeq platform and the NovaSeq 6000 SP Reagent kit (Illumina, San Diego, CA, USA, Cat. No.: 20028402). Sequence data analyses were mainly performed using QIIME2 and R packages (v3.2.0). All sequence data have been submitted to the NCBI under BioProject ID PRJNA1121036.

### 2.10. Statistical Analyses

The effects of the S, Q, and their interaction on the apparent indicators were assessed using analysis of variance (ANOVA) and the general linear model (GLM) procedure using IBM SPSS Statistics 19.0 (IBM Corp., Armonk, NY, USA). Multiple mean comparisons were performed using one-way ANOVA and Tukey’s multiple-range test. All means were considered statistically significant at *p* < 0.05. DESeq was employed to analyze the differentially expressed genes (DEGs) in transcriptomics, with DEGs screened based on a fold change (FC) > 2 or <0.5, and *p* < 0.05. The microbial phylum, family, and genus in the cecum were compared using the Wilcoxon rank-sum test, while cecal microbial species were compared using linear discriminant analysis effect size (LEfSe) analysis. The current threshold for the linear discriminant analysis (LDA) was set at 2.

## 3. Results

### 3.1. Growth Performance 

The OS treatment did not affect (*p* > 0.05) the ADG, ADFI, F:G, and final body weights (FBWs) of the rats. The Q supplementation decreased (*p* < 0.05) the ADFI during 1–14 days and 1–28 days (Table 2). The growth performance was not influenced by the interaction between the soybean meal types and quercetin levels (S × Q).

### 3.2. Relative Organ Weight

The OS treatment decreased (*p* < 0.05) the relative weights of the pancreas, stomach, and cecum (Table 3). S × Q exerted a significant effect on the relative weight of the duodenum (*p* = 0.019).

### 3.3. Lengths of Intestinal Tissues

The lengths of the jejunum, ileum, cecum, and colorectum were increased (*p* < 0.01) by the OS treatment; meanwhile, that of the duodenum was also increased (*p* < 0.05) by the Q supplementation (Table 4). S × Q exerted a significant effect on the length of the colorectum (*p* = 0.002).

### 3.4. Intestinal Morphology

**Figure 1:**
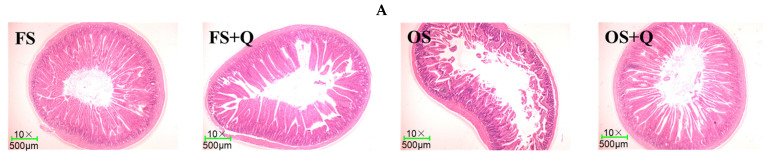

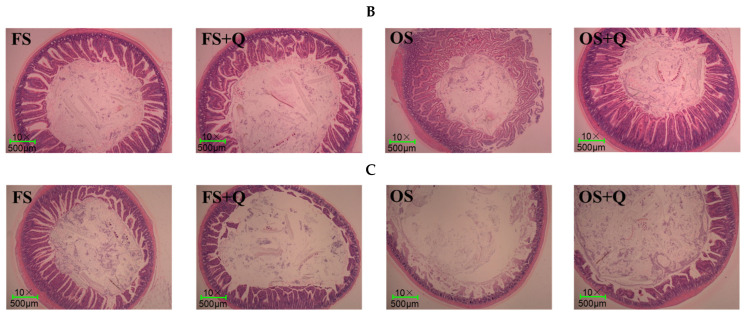
The intestinal morphology of rats. FS: fresh soybean meal; FS + Q: fresh soybean meal + quercetin; OS: protein-oxidized soybean meal; OS + Q: protein-oxidized soybean meal + quercetin. (**A**) Duodenum. (**B**) Jejunum. (**C**) Ileum.

As presented in Table 5 and Figure 1, the OS treatment decreased (*p* < 0.05) the villus height and crypt depth of the duodenum. Conversely, the Q supplementation increased (*p* < 0.05) the villus height of the ileum. Moreover, S × Q exerted significant effects on the villus heights of the duodenum (*p* = 0.036) and ileum (*p* < 0.001) and the villus height-to-crypt depth ratios of the jejunum (*p* = 0.046) and ileum (*p* = 0.024), which showed that OS + Q reversed the OS-induced decrease in the villus heights of the duodenum and ileum.

### 3.5. Digestive Enzyme Activity

As shown in Table 6, the activities of the pancreatic trypsin, jejunal trypsin, pancreatic α-amylase, and jejunal α-amylase were increased (*p* < 0.05) by the OS treatment, while those of the pancreatic lipase and jejunal lipase were decreased (*p* < 0.05). The Q supplementation exhibited a positive effect on the activities of the pancreatic lipase and jejunal lipase (*p* < 0.05). S × Q exerted a significant effect on the pancreatic lipase activity (*p* = 0.023), and OS + Q reversed the OS-induced decrease in it. 

### 3.6. Nutrient Apparent Digestibility 

As shown in Table 7, the OS treatment exhibited a negative effect (*p* < 0.05) on the AID of 17 common AAs (excluding cysteine), while the Q supplementation exhibited a positive effect (*p* < 0.05) on that of 17 common AAs (excluding aspartic acid and cysteine). The S × Q revealed that OS + Q reversed (*p* < 0.05) the OS-induced decrease in the AID of 17 common AAs (excluding aspartic acid and cysteine). 

As shown in Table 8, from the principal effects analysis, the OS treatment increased (*p* < 0.001) the ATTD of DM and CP, and the addition of Q also enhanced (*p* < 0.05) that of CP and EE. S × Q exerted significant effects on the ATTD of DM (*p* < 0.001), CP (*p* < 0.001), and EE (*p* < 0.001); but, from the one-way ANOVA, compared with the FS group, the ATTD of DM, CP, and EE in the OS group decreased (*p* < 0.05), and the ATTD of DM and CP in the OS + Q group increased (*p* < 0.05). Combining these two analyses, the OS + Q reversed (*p* < 0.05) the OS-induced decreases in the ATTD of DM, CP, and EE.

### 3.7. Transcriptomics Analysis

**Figure 2:**
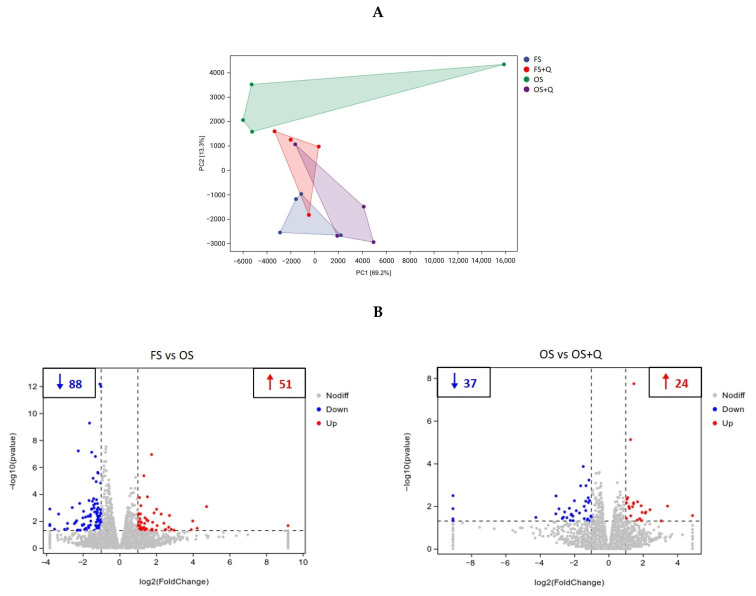

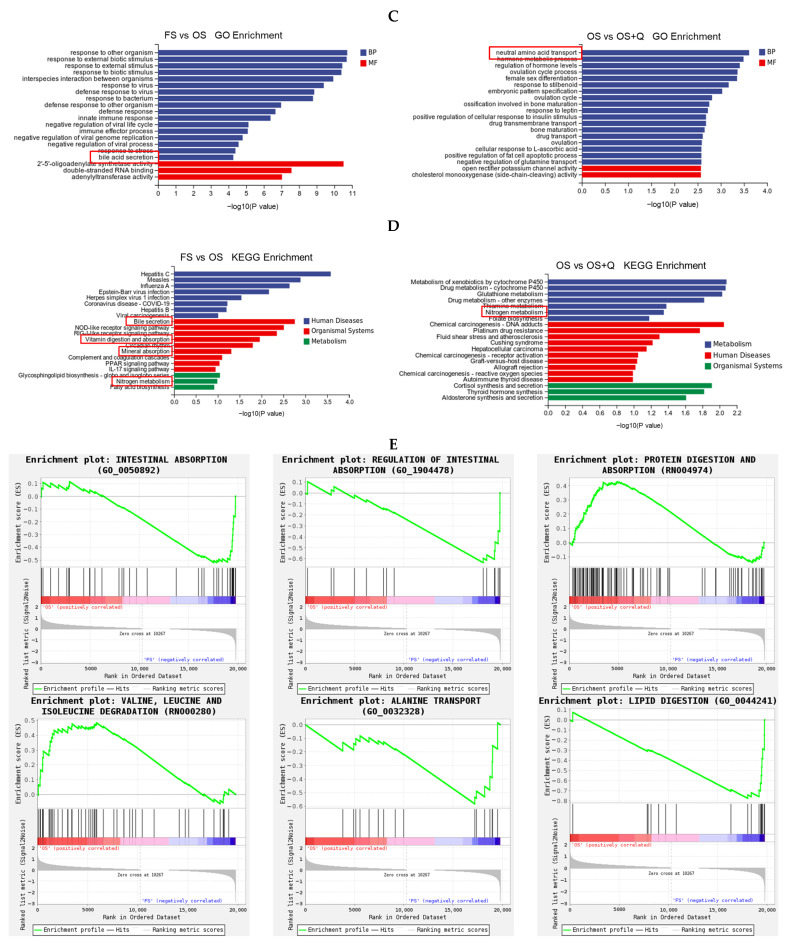

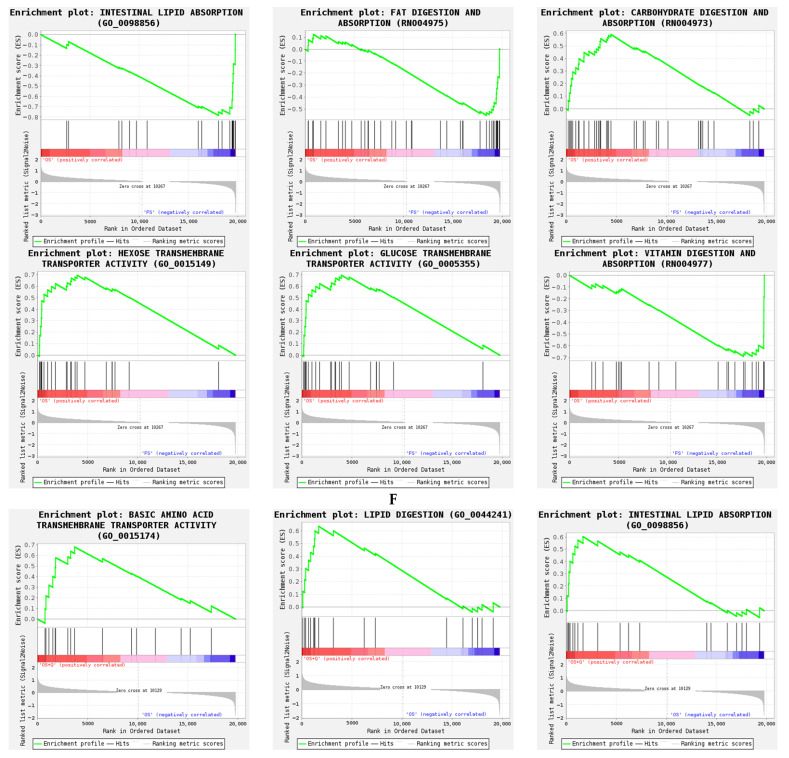
Transcriptome analysis of jejunum. FS: fresh soybean meal; FS + Q: fresh soybean meal + quercetin; OS: protein-oxidized soybean meal; OS + Q: protein-oxidized soybean meal + quercetin. *n* = 4 for each group. (**A**) Principal component analysis (PCA) of transcriptional profiling of the rat jejunum tissues among four groups. (**B**) Differential gene expression analysis. Red dots (up) represent significantly upregulated genes (*p* < 0.05, FC ≥ 2); green dots (down) represent significantly downregulated genes (*p* < 0.05, FC ≤ 0.5); and gray dots (no) represent insignificant DEGs. (**C**) GO pathway enrichment analysis of top 20 pathways. The pathways with the red box are related to the intestinal digestive and absorptive functions. (**D**) KEGG pathway enrichment analysis of top 20 pathways. The pathways with the red box are related to the intestinal digestive and absorptive functions. (**E**) Significant pathways of GSEA analysis results in FS vs. OS comparison groups (nominal *p* < 0.05). (**F**) Significant pathways of GSEA analysis results in OS vs. OS + Q comparison groups (nominal *p* < 0.05).

Principal component analysis (PCA) showed a distinct separation between the OS group and the FS, FS + Q, and OS + Q groups (Figure 2A). Volcano plots showed 139 genes between the FS and OS groups (51 upregulated genes, 88 downregulated genes), and 61 genes between the OS and OS + Q groups (24 upregulated genes, 37 downregulated genes) (Figure 2B). Gene Ontology (GO) enrichment analysis revealed that the top 20 significantly enriched GO terms in the biological process (BP) and molecular function (MF), such as “bile acid secretion” between the FS and OS groups, and “neutral amino acid transport” between the OS and OS + Q groups, were related to the intestinal digestive and absorptive functions (Figure 2C). Kyoto Encyclopedia of Genes and Genomes (KEGG) enrichment analysis showed that the top 20 significant pathways, including “bile secretion”, “vitamin digestion and absorption”, “mineral absorption”, and “nitrogen metabolism” between the FS and OS groups, and “nitrogen metabolism” between the OS and OS + Q groups, were related to the intestinal digestive and absorptive functions (Figure 2D). To be able to systematize the gene expression results, we performed gene set enrichment analysis (GSEA) based on all the annotated genes to determine the regulation of the OS treatment and Q supplementation on significant pathways. Compared with the FS group, the “intestinal absorption”, “regulation of intestinal absorption”, “alanine transport”, “lipid digestion”, “intestinal lipid absorption”, “fat digestion and absorption”, and “vitamin digestion and absorption” in the OS group were downregulated (nominal *p* < 0.05), while the “protein digestion and absorption”, “valine, leucine, and isoleucine degradation”, “hexose transmembrane transporter activity”, “glucose transmembrane transporter activity”, and “carbohydrate digestion and absorption” in the OS group were upregulated (nominal *p* < 0.05) (Figure 2E). However, the “basic amino acid transmembrane transporter activity”, “lipid digestion”, and “intestinal lipid absorption” were positively regulated in the OS + Q group compared with the OS group (nominal *p* < 0.05) (Figure 2F).

### 3.8. Microbiomics Analysis 

**Figure 3:**
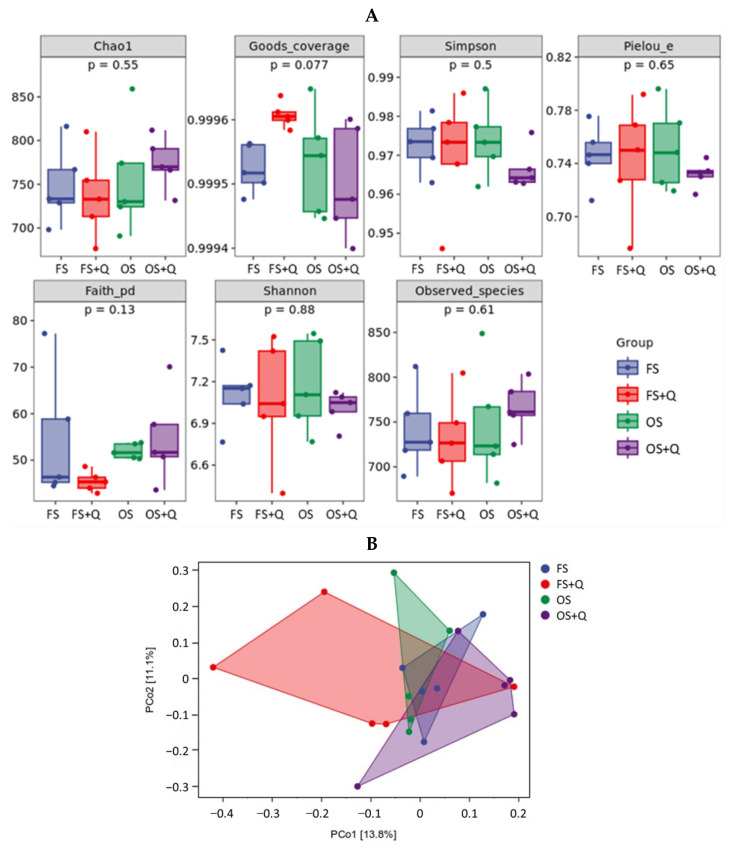

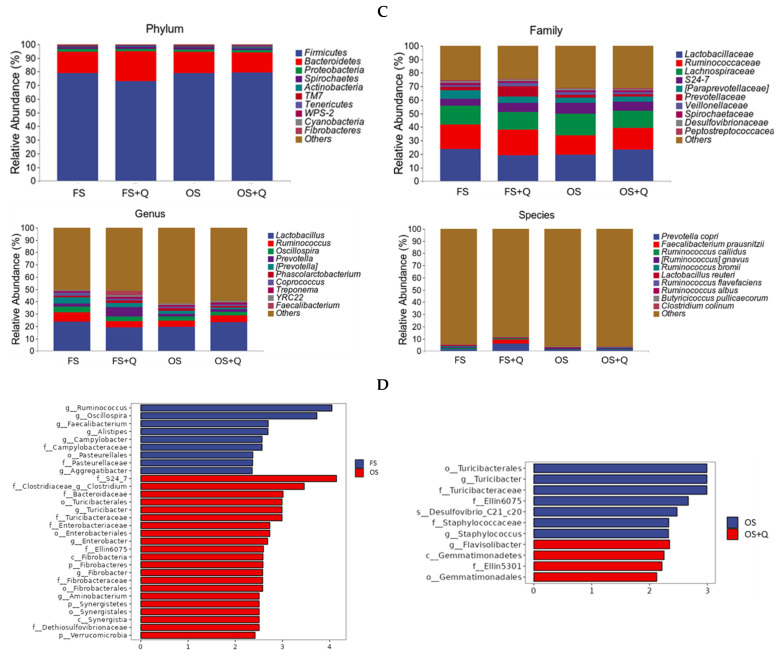
Microbiomics analysis of cecal digesta. FS: fresh soybean meal; FS + Q: fresh soybean meal + quercetin; OS: protein-oxidized soybean meal; OS + Q: protein-oxidized soybean meal + quercetin. *n* = 5 for each group. (**A**) Changes in the α-diversity of cecal microbiota communities, as indicated by the chao1, goods_coverage, simpson, pielou_e, faith_pd, shannon, and observed_species indices. (**B**) PCoA of cecal microbiota. (**C**) The relative abundances of cecal microbiota at the phylum, family, genus, and species levels. (**D**) LDA scores.

High-throughput 16S rRNA sequencing was performed to determine the effect of the OS treatment and Q supplementation on the cecal microbiome of the rats. None of the α-diversity indices were significantly influenced (*p* > 0.05) by the OS treatment and Q supplementation (Figure 3A). Principal Coordinate Analysis (PCoA) showed that the different treatments induced the distinct (*p* = 0.017) clustering of bacterial communities (Figure 3B). At the phylum level (top 10), the OS treatment increased the relative abundances of *Actinobacteria* (*p* = 0.009) and *Fibrobacteres* (*p* < 0.001). At the family level (top 10), the OS treatment decreased the relative abundance of *Ruminococcaceae* (*p* = 0.021). At the genus level (top 10), the OS treatment decreased the relative abundance of *Oscillospira* (*p* = 0.015). At the species level (top 10), the Q supplementation decreased the relative abundance of *[Ruminococcus] gnavus* (*p* = 0.025) (Figure 3C). LEfSe analysis was performed to identify taxonomic biomarkers in the cecal microbiota. In the FS vs. OS comparison groups, the FS treatment increased the relative abundances of 13 kinds of bacteria with different levels, such as *Ruminococcus* (genus), while the OS treatment increased the relative abundances of 24 kinds of bacteria with different levels, such as *S24_ 7* (family). In the OS vs. OS + Q comparison groups, the OS treatment increased the relative abundances of seven kinds of bacteria with different levels, such as *Turicibacterales* (order), and the OS + Q treatment increased the relative abundances of four kinds of bacteria with different levels, such as *Flavisolibacter* (genus) (Figure 3D).

## 4. Discussion

Although the ADG of the rats was not significantly altered (*p* > 0.05) by the OS treatment, there was a tendency for an increase in the ADFI (*p* = 0.099) from d 15 to 28, which was similar to the previous finding that OS could increase the ADFI in broilers, but not significantly [7]. Moreover, the Q supplementation resulted in a decrease in the ADFI (*p* < 0.05) in the rats, which was in line with the findings of previous studies [24], and this effect may be attributed to the bitter taste of Q [25]. 

On the one hand, the OS treatment reduced (*p* < 0.05) the relative weights of the pancreas, stomach, and cecum, which was consistent with previous findings that diets containing heat-oxidized SPI decreased the relative weights of the duodenum and jejunum in broilers [6]. Moreover, the OS treatment reduced (*p* < 0.05) the villus height of the duodenum in the rats, which was consistent with previous findings that oxidized vegetable oil decreased (*p* = 0.001) the villus height of the duodenum in broilers. On the other hand, the OS treatment increased the lengths of the jejunum, ileum, cecum, and colorectum in the rats. Generally speaking, the main causes of intestinal elongation in animals include the dilution of easily digestible compounds with indigestible material [26]. So, the intestinal elongation of the rats may be attributed to the indigestible properties of OS [6]. Moreover, OS + Q reversed (*p* < 0.05) the OS-induced decreases in the length of the duodenum and the villus heights of the duodenum and ileum of the rats. Overall, the above results suggested that adding Q to the OS diet can ameliorate the negative effects of OS on the growth and development of the digestive organs in rats.

It is well known that nutrient digestibility depends not only on the relative weights, lengths, and morphologies of the digestive organs, but also on the digestive enzyme activities and the number of intestinal transport carriers. In the present study, the OS treatment increased the activities of pancreatic trypsin, jejunal trypsin, pancreatic α-amylase, and jejunal α-amylase. However, other experiments have indicated that the activities of trypsin and α-amylase were all reduced in broilers fed heat-oxidized SPI [6] or a heat-induced OS diet [7]. These inconsistencies may be related to the differences in the digestive systems between rats and broilers. The AID of 17 common AAs (excluding cysteine) were reduced by the OS treatment, which was consistent with a previous study that showed that the AID of all AAs was reduced by autoclaved-induced OS in growing pigs [27]. From the principal effects analysis, the OS treatment increased (*p* < 0.001) the ATTD of DM and CP; however, from the one-way ANOVA, the ATTD of DM, CP, and EE were all decreased (*p* < 0.05) in the OS group compared with the FS group, which was in line with previous studies that found that the apparent total digestibility of CP and DM were all reduced in broilers fed heat-oxidized SPI [6] or a heat-induced OS diet [7]. Moreover, OS + Q reversed (*p* < 0.05) the OS-induced decrease in the activity of lipase, the AID of 17 common AAs (excluding aspartic acid and cysteine), and the ATTD of DM, CP, and EE. These results may be because Q has beneficial impacts on regulating protein and lipid metabolism [13,14] and the mRNA expression of nutritional transporters [11].

**Figure A1:**
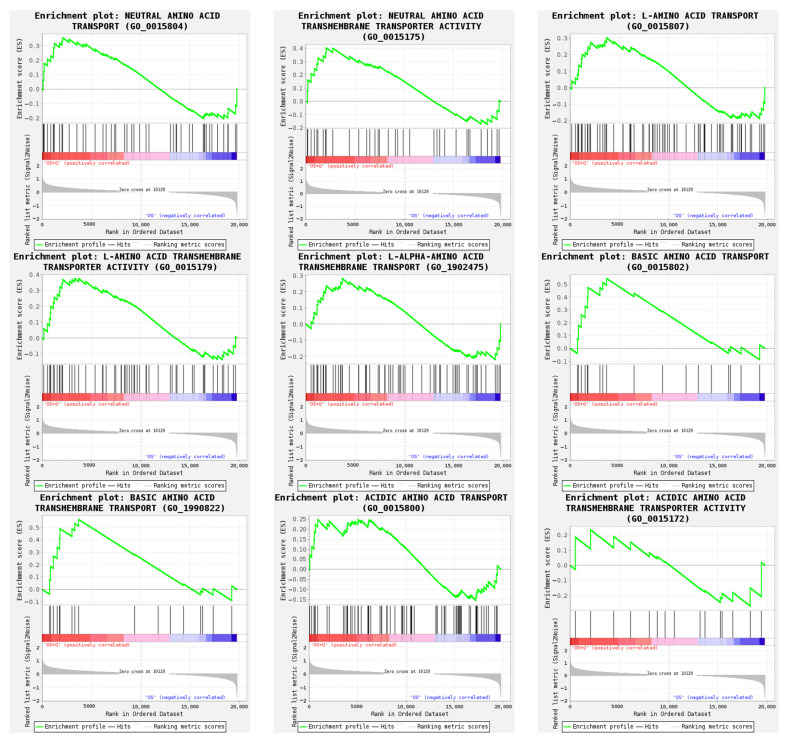
Non-significant pathways of GSEA analysis results in OS vs. OS + Q comparison groups (nominal *p* > 0.05).

Transcriptomic analysis indicated that the OS treatment positively regulated the significant pathways of “protein digestion and absorption” and “valine, leucine, and isoleucine degradation”, but it negatively regulated the significant pathways of “intestinal absorption”, “regulation of intestinal absorption”, and “alanine transport” compared with the FS treatment, which were in agreement with previous research that found that heat-induced OS decreased the mRNA expression levels of amino acid transporters and peptide transporters in the intestinal mucosa of laying hens [8]. These results explain why the OS treatment reduced the AID of 17 common AAs (excluding cysteine) and the ATTD of CP in this study. As we know, it is crucial to ensure that all amino acids in the diet are fully digested and absorbed by the small intestine as much as possible [28] because elevated levels of undigested protein can lead to an escalation in pathogenic microorganisms and are linked to a heightened risk of metabolic diseases [29]. Therefore, enhancing the digestion and absorption of proteins and AAs in the small intestine through nutritional strategies is imperative. Based on the gene set enrichment analysis (GSEA) results, the significant pathway of “basic amino acid transmembrane transporters” and other non-significant (nominal *p* > 0.05) pathways of AA transport (Figure A1) were all positively regulated in the OS + Q group compared with the OS group. These results revealed that the OS + Q treatment almost completely reversed the AID of AAs, restoring it to the FS treatment levels. 

Moreover, the OS treatment downregulated the significant pathways of “lipid digestion”, “intestinal lipid absorption”, “fat digestion and absorption”, and “vitamin digestion and absorption”, which was consistent with the decreases in the relative weight of the pancreas, duodenal villi height, lipase activity, and ATTD of EE in this study. A previous study also indicated that heat-oxidized SPI inconspicuously decreased (*p* > 0.05) the lipase activity and the ATTD of EE in broilers [6]. The OS treatment reduced the relative weights of the pancreas, duodenal villi height, and lipase activity and downregulated the pathways of “intestinal absorption” and “regulation of intestinal absorption”, thereby reducing the entire digestive and absorptive functions of the intestine. So, the intestinal digestion and absorption of nutrients such as lipids and vitamins were both inhibited. However, the absorption of carbohydrates is mainly through passive absorption, which does not consume energy and does not require complex packaging and transport processes. So, with the increases in α-amylase activity, the significant pathways of “hexose transmembrane transporter activity”, “glucose transmembrane transporter activity”, and “carbohydrate digestion and absorption” were upregulated by the OS treatment. Moreover, the significant pathways of “lipid digestion” and “intestinal lipid absorption” were positively regulated in the OS + Q group compared with the OS group. So far, from the perspective of jejunal gene transcription, the OS + Q treatment effectively addressed the issue of reduced nutrient digestion and absorption in the rat small intestine induced by the OS treatment.

The trade-off between saccharolytic and proteolytic fermentation by the gut microbiota is a prominent subject of discussion, particularly in response to alterations in the quality and quantity of dietary carbohydrates and proteins [30]. Additionally, the detrimental effects of oxidized proteins on the gut microbiota are closely linked to intestinal barrier damage and the induction of an inflammatory response [31]. In the present study, the species composition analysis results of the cecal microbiomics FS vs. OS comparison groups showed that the OS treatment increased the relative abundances of *Actinobacteria* (phylum) (*p* = 0.009) and *Fibrobacteres* (phylum) (*p* < 0.001) and decreased the abundances of *Ruminococcaceae* (family) (*p* = 0.021) and *Oscillospira* (genus) (*p* = 0.015). It is well known that *Actinobacteria* (phylum) [32], *Fibrobacteres* (phylum), and *Ruminococcaceae* (family) [33] are mainly involved in the biodegradation of cellulose and resistant starch, which suggests that there is both an increase and a decrease in bacterial abundance related to carbohydrate degradation. As for *Actinobacteria* (phylum), several studies have reported that it is related to protein post-translational modification and protein degradation [34], and it also plays a special role in the protein degradation of OS. As for *Oscillospira* (genus), several studies have summarized that the abundance of *Oscillospira* (genus) is highly negatively correlated with ulcerative colitis [35,36], obesity, and obesity-related chronic inflammation [37]. In the present study, a significantly reduced abundance of *Oscillospira* (genus) predicted that the OS treatment increased the risk of intestinal and metabolic diseases in the rats. In contrast, the species composition analysis results of the cecal microbiomics OS vs. OS + Q comparison groups showed that the OS + Q treatment decreased the relative abundance of *[Ruminococcus] gnavus* (species) (*p* = 0.025), whereas the elevated relative abundance of *Ruminococcus gnavus* (species) is strongly associated with enteritis symptoms and has been identified as one of the causes of Crohn’s disease symptoms [38], and it was shown that OS + Q treatment decreased the risk of Crohn’s disease in rats. 

Finally, the results of the LEfSe analysis of the cecal microbiomics showed the same findings. In the FS vs. OS comparison groups, the OS treatment increased the LDA scores of *Bacteroidaceae* (family) [39,40,41], *Turicibacter* (genus) [42], *Aminobacterium* (genus) [43], and *Dethiosulfovibrionaceae* (family) [44], whereas these bacteria can degrade proteins and ferment amino acids, which was associated with an increase in the ATTD of CP in the rats. However, several studies have been reported that *Turicibacter* (genus) is positively associated with colitis [45], *Enterobacteriaceae* (family) is also a potentially harmful bacteria [46], and *Dethiosulfovibrionaceae* is related to Behcet’s disease (BD) [47], and it can be understood that the OS treatment increased the risk of intestinal diseases in the rats. In the OS vs. OS + Q comparison groups, the OS + Q treatment increased the LDA scores of *Flavisolibacter* (genus) and *Gemmatimonadetes* (class). *Flavisolibacter* (genus) has been found to have the functions of improving nutrient digestion and absorption and promoting animal growth [48]. *Gemmatimonadetes* (class) is involved in the regulation of the immune function and nutrient metabolism of animal hosts [49]. Thus far, the increase in the abundances of these bacteria indicates that OS + Q treatment may enhance or partially enhance nutrient digestion, absorption, and metabolism and mitigate intestinal diseases resulting from OS treatment.

## 5. Conclusions

In conclusion, the OS treatment decreased the relative weights of the pancreas, stomach, and cecum, duodenal villi height, and lipase activity, thereby reducing the AID of 17 common AAs (excluding cysteine) and the ATTD of DM, CP, and EE in the rats, while the OS + Q treatment increased the duodenal length, ileal villus height, and pancreatic lipase activity, thereby reversing the decreases in the AID of 17 amino acids (excluding aspartic acid and cysteine) and the ATTD of DM, CP, and EE. Transcriptomics analysis revealed that the OS treatment downregulated the pathways of “intestinal absorption”, “alanine transport”, “lipid digestion and absorption”, and “vitamin digestion and absorption”; while the OS + Q treatment upregulated the pathways of “basic amino acid transmembrane transporter activity” and “lipid digestion and absorption”. Microbiomics analysis revealed that the OS treatment increased (*p* < 0.05) the relative abundances of protein-degrading and inflammation-triggering bacteria in the cecum, while the relative abundances of beneficial bacteria were elevated (*p* < 0.05) by the OS + Q treatment. The results of this study reveal the beneficial effects of Q on improving the intestinal digestive and absorptive functions and microbial composition in rats fed OS.

## Figures and Tables

**Table 1:** Ingredient compositions and nutrient levels of basal diets ^1^ (g/kg, as-fed basis).

Items	FS ^2^	OS ^2^
Ingredients		
Corn	375.87	375.87
Subflour	19.94	19.94
Wheat	199.4	199.4
Fresh soybean meal	299.1	-
Protein-oxidized soybean meal	-	299.1
Wheat bran	9.97	9.97
Soybean oil	19.94	19.94
Limestone	11.96	11.96
Dicalcium phosphate	14.96	14.96
Sodium chloride	2.49	2.49
Magnesium oxide	1.5	1.5
Choline chloride	1.99	1.99
Premix ^3^	39.88	39.88
Cr_2_O_3_	3	3
Nutrient levels ^4^		
Crude protein	209.37	209.37
ME, MJ/kg	133.8	133.8
Crude lipid	44.87	44.87
Crude fiber	39.88	39.88
Crude ash	57.83	57.83
Calcium	10.97	10.97
Total phosphorus	7.48	7.48
Lysine	12.96	12.96
Methionine + cystine	8.57	8.57

^1^ The quercetin-added contrast diets consisted of adding 400 mg quercetin premix per kilogram of corresponding basal diets; ^2^ FS: fresh soybean meal-based diets; OS: protein-oxidized soybean meal-based diets. ^3^ The premix provided the following per kilogram of diets: arginine, 0.04 g; histidine, 0.02 g; isoleucine, 1.98 g; leucine, 1.24 g; lysine, 4.05 g; methionine, 2.39 g; cystine, 0.02 g; phenylalanine, 0.03 g; tyrosine, 0.02 g; threonine, 1.69 g; tryptophan, 0.8 g; valine, 1.63 g; Na, 0.82 g; Mg, 0.02 g; K, 0.03 g; Fe, 99.85 mg; Cu, 7.21 mg; Mn, 43.88 mg; Zn, 59.88 mg; I, 0.67 mg; Se, 0.2 mg; carotene, 0.01 mg; vitamin E, 103.3 IU; thiamine, 41.44 mg; riboflavin, 18.65 mg; pantothenic acid, 37.19 mg; nicotinic acid, 92.36 mg; biotin, 0.32 mg; folic acid, 10.05 mg; choline, 5.86 mg; pyridoxine hydrochloride, 19.24 mg; cyanocobalamin, 0.04 mg; linoleic acid, 0.08 g; vitamin A, 21,705 IU; vitamin D, 3157 IU; vitamin K, 8.64 mg. ^4^ Nutrient levels are calculated values.

**Table 2:** Effects of S, Q, and their interaction on the growth performance of rats.

Item	Group				SEM	*p*-Value		
	FS	FS + Q	OS	OS + Q		S	Q	S × Q
1–14 d								
ADG (g/d)	8.99	8.81	8.84	8.79	0.09	0.645	0.512	0.712
ADFI (g/d)	21.39	20.65	21.18	20.78	0.14	0.881	0.046	0.516
F:G (g:g)	2.38	2.35	2.40	2.36	0.02	0.621	0.384	0.959
15–28 d								
ADG (g/d)	7.53	7.52	7.85	7.58	0.09	0.297	0.449	0.468
ADFI (g/d)	23.49	23.12	24.64	23.41	0.23	0.099	0.070	0.307
F:G (g:g)	3.12	3.08	3.14	3.09	0.02	0.749	0.356	0.968
1–28 d								
ADG (g/d)	8.26	8.17	8.35	8.19	0.06	0.677	0.325	0.800
ADFI (g/d)	22.44	21.89	22.91	22.10	0.17	0.284	0.044	0.677
F:G (g:g)	2.72	2.68	2.75	2.70	0.02	0.451	0.212	0.838
FBW (g)	350.45	344.98	347.00	341.85	2.02	0.424	0.200	0.970

S: soybean meal types; Q: quercetin levels; FS: fresh soybean meal; FS + Q: fresh soybean meal + quercetin; OS: protein-oxidized soybean meal; OS + Q: protein-oxidized soybean meal + quercetin; SEM: standard error of means (*n* = 12); S × Q: interaction between soybean meal types and quercetin levels; FBW: final body weight.

**Table 3:** Effects of S, Q, and their interaction on the relative organ weights of rats (%).

Item	Group				SEM	*p*-Value		
	FS	FS + Q	OS	OS + Q		S	Q	S × Q
Pancreas	0.61	0.62	0.47	0.47	0.03	0.044	0.917	0.931
Liver	4.42	4.31	4.48	4.46	0.04	0.170	0.399	0.538
Stomach	0.45	0.44	0.42	0.42	0.01	0.027	0.674	0.405
Duodenum	0.56 ^a^	0.53 ^ab^	0.44 ^b^	0.56 ^a^	0.02	0.104	0.105	0.019
Jejunum	0.56	0.64	0.59	0.60	0.01	0.848	0.089	0.137
Ileum	1.15	1.18	1.15	1.12	0.02	0.269	0.992	0.341
Cecum	0.35	0.34	0.33	0.32	0.01	0.032	0.192	0.878
Colorectum	0.41	0.41	0.41	0.42	0.01	0.656	0.287	0.714

^a,b^ Within a row, means without a common superscript differ significantly (*p* < 0.05). S: soybean meal types; Q: quercetin levels; FS: fresh soybean meal; FS + Q: fresh soybean meal + quercetin; OS: protein-oxidized soybean meal; OS + Q: protein-oxidized soybean meal + quercetin; SEM: standard error of means (*n* = 12); S × Q: interaction between soybean meal types and quercetin levels.

**Table 4:** Effects of S, Q, and their interaction on the lengths of the intestinal tissues of rats (cm).

Item	Group				SEM	*p*-Value		
	FS	FS + Q	OS	OS + Q		S	Q	S × Q
Duodenum	24.32	24.83	22.98	26.13	0.44	0.977	0.037	0.130
Jejunum	28.25 ^c^	29.75 ^bc^	32.94 ^a^	31.58 ^ab^	0.45	<0.001	0.927	0.068
Ileum	56.85 ^b^	59.45 ^ab^	65.08 ^a^	64.04 ^a^	0.89	<0.001	0.617	0.245
Cecum	6.29 ^b^	6.68 ^ab^	7.25 ^ab^	7.53 ^a^	0.14	0.001	0.188	0.840
Colorectum	13.08 ^b^	15.92 ^a^	17.73 ^a^	16.67 ^a^	0.38	<0.001	0.148	0.002

^a,b,c^ Within a row, means without a common superscript differ significantly (*p* < 0.05). S: soybean meal types; Q: quercetin levels; FS: fresh soybean meal; FS + Q: fresh soybean meal + quercetin; OS: protein-oxidized soybean meal; OS + Q: protein-oxidized soybean meal + quercetin; SEM: standard error of means (*n* = 12); S × Q: interaction between soybean meal types and quercetin levels.

**Table 5:** Effects of S, Q, and their interaction on the intestinal morphology of rats.

Item	Group				SEM	*p*-Value		
	FS	FS + Q	OS	OS + Q		S	Q	S × Q
Villus height (μm)								
Duodenum	641.04 ^a^	621.72 ^a^	437.72 ^b^	571.79 ^a^	20.24	0.001	0.114	0.036
Jejunum	466.75	458.63	488.99	532.11	13.40	0.075	0.511	0.336
Ileum	293.89 ^ab^	272.48 ^bc^	208.51 ^c^	352.57 ^a^	10.77	0.879	0.001	<0.001
Crypt depth (μm)								
Duodenum	200.78 ^ab^	218.83 ^a^	173.59 ^b^	198.42 ^ab^	6.03	0.045	0.070	0.771
Jejunum	161.73	173.71	185.98	165.70	6.43	0.534	0.750	0.219
Ileum	148.98	147.53	126.58	163.44	7.63	0.832	0.251	0.215
V:C ratio								
Duodenum	3.18	2.92	2.66	3.07	0.11	0.395	0.726	0.127
Jejunum	3.09	2.77	2.82	3.35	0.11	0.463	0.625	0.046
Ileum	2.28	2.03	1.71	2.55	0.12	0.912	0.213	0.024

^a,b,c^ Within a row, means without a common superscript differ significantly (*p* < 0.05). S: soybean meal types; Q: quercetin levels; FS: fresh soybean meal; FS + Q: fresh soybean meal + quercetin; OS: protein-oxidized soybean meal; OS + Q: protein-oxidized soybean meal + quercetin; SEM: standard error of means (*n* = 12); S × Q: interaction between soybean meal types and quercetin levels; V:C: villus height to crypt depth.

**Table 6:** Effects of S, Q, and their interaction on the digestive enzyme activities of rats.

Items	Group				SEM	*p*-Value		
	FS	FS + Q	OS	OS + Q		S	Q	S × Q
Pancreatic trypsin (U/mg prot.)	1202.71 ^b^	1191.17 ^b^	1437.3 ^ab^	1567.76 ^a^	52.25	0.001	0.424	0.342
Jejunal trypsin (U/mg prot.)	514.90	529.73	670.05	726.14	34.86	0.010	0.552	0.728
Pancreatic lipase (U/g prot.)	46.59 ^a^	46.36 ^a^	41.29 ^b^	46.71 ^a^	0.76	0.041	0.034	0.023
Jejunal lipase (U/g prot.)	26.60 ^a^	27.61 ^a^	21.39 ^b^	25.05 ^ab^	0.74	0.001	0.027	0.179
Pancreatic α-amylase (U/mg prot.)	7.50 ^c^	7.94 ^bc^	9.20 ^a^	8.86 ^ab^	0.22	0.001	0.873	0.204
Jejunal α-amylase (U/mg prot.)	1.29 ^b^	1.36 ^b^	2.22 ^a^	2.00 ^a^	0.12	<0.001	0.628	0.367

^a,b,c^ Within a row, means without a common superscript differ significantly (*p* < 0.05). S: soybean meal types; Q: quercetin levels; FS: fresh soybean meal; FS + Q: fresh soybean meal + quercetin; OS: protein-oxidized soybean meal; OS + Q: protein-oxidized soybean meal + quercetin; SEM: standard error of means (*n* = 4); S × Q: interaction between soybean meal types and quercetin levels.

**Table 7:** Effects of S, Q, and their interaction on the AID of AAs in rats.

Item	Group				SEM	*p*-Value		
	FS	FS + Q	OS	OS + Q		S	Q	S × Q
Alanine	71.70 ^a^	71.18 ^a^	64.47 ^b^	71.75 ^a^	0.95	<0.001	<0.001	<0.001
Arginine	80.79 ^a^	80.23 ^a^	73.08 ^b^	80.03 ^a^	0.97	<0.001	<0.001	<0.001
Aspartic acid	67.21 ^a^	65.06 ^a^	61.11 ^b^	65.93 ^a^	0.74	0.003	0.063	<0.001
Cysteine	77.60 ^a^	64.90 ^b^	67.30 ^ab^	69.51 ^ab^	1.81	0.298	0.074	0.019
Glutamic acid	78.89 ^a^	78.00 ^a^	73.70 ^b^	78.80 ^a^	0.66	<0.001	0.001	<0.001
Glycine	61.23 ^a^	58.40 ^a^	47.57 ^b^	58.77 ^a^	1.61	<0.001	<0.001	<0.001
Histidine	74.29 ^a^	72.23 ^a^	67.25 ^b^	73.93 ^a^	0.91	0.008	0.017	<0.001
Isoleucine	77.84 ^a^	77.26 ^a^	72.79 ^b^	78.22 ^a^	0.69	0.002	0.001	<0.001
Leucine	76.17 ^a^	75.42 ^a^	70.35 ^b^	76.55 ^a^	0.78	0.001	<0.001	<0.001
Lysine	74.55 ^a^	74.28 ^a^	68.85 ^b^	75.76 ^a^	0.84	0.006	<0.001	<0.001
Methionine	88.91 ^b^	89.40 ^b^	84.83 ^c^	91.59 ^a^	0.76	0.043	<0.001	<0.001
Phenylalanine	75.89 ^a^	74.88 ^a^	69.84 ^b^	76.36 ^a^	0.81	0.002	0.001	<0.001
Proline	76.60 ^a^	75.24 ^a^	70.70 ^b^	75.80 ^a^	0.71	<0.001	0.002	<0.001
Serine	69.34 ^a^	67.55 ^a^	62.88 ^b^	68.72 ^a^	0.79	0.001	0.003	<0.001
Threonine	65.39 ^a^	62.64 ^a^	57.73 ^b^	64.30 ^a^	0.93	0.002	0.018	<0.001
Tyrosine	74.39 ^a^	73.46 ^a^	66.63 ^b^	73.27 ^a^	0.95	<0.001	<0.001	<0.001
Valine	73.97 ^a^	72.47 ^a^	68.61 ^b^	73.04 ^a^	0.66	0.002	0.029	0.001
Total AAs	74.44 ^a^	73.10 ^a^	68.01 ^b^	74.03 ^a^	0.81	0.001	0.002	<0.001

^a,b,c^ Within a row, means without a common superscript differ significantly (*p* < 0.05). S: soybean meal types; Q: quercetin levels; FS: fresh soybean meal; FS + Q: fresh soybean meal + quercetin; OS: protein-oxidized soybean meal; OS + Q: protein-oxidized soybean meal + quercetin; SEM: standard error of means (*n* = 3); S × Q: interaction between soybean meal types and quercetin levels. The nutrient apparent digestibility was calculated in dry matter form.

**Table 8:** Effects of S, Q, and their interaction on the ATTD of DM, CP, and EE in rats.

Item	Group				SEM	*p*-Value		
	FS	FS + Q	OS	OS + Q		S	Q	S × Q
DM	87.40 ^b^	86.07 ^d^	87.26 ^c^	88.37 ^a^	0.25	<0.001	<0.001	<0.001
CP	84.78 ^b^	84.09 ^c^	84.23 ^c^	86.51 ^a^	0.29	<0.001	<0.001	<0.001
EE	92.07 ^ab^	91.04 ^bc^	89.86 ^c^	92.49 ^a^	0.33	0.210	0.021	<0.001

^a,b,c,d^ Within a row, means without a common superscript differ significantly (*p* < 0.05). S: soybean meal types; Q: quercetin levels; FS: fresh soybean meal; FS + Q: fresh soybean meal + quercetin; OS: protein-oxidized soybean meal; OS + Q: protein-oxidized soybean meal + quercetin; SEM: standard error of means (*n* = 3); S × Q: interaction between soybean meal types and quercetin levels. The nutrient apparent digestibility was calculated in dry matter form.

## Data Availability

The original contributions presented in this study is included in the article, and further inquiries can be directed to the first author or corresponding author.

## Abbreviations

S	soybean meal	ADFI	average daily feed intake
FS	fresh soybean meal	F:G	feed conversion ratio
OS	protein-oxidized soybean meal	SD	Sprague–Dawley
Q	quercetin	SPF	specific pathogen-free
SPI	soy protein isolate	PCA	principal component analysis
AID	apparent ileal digestibility	GO	Gene Ontology
ATTD	apparent total tract digestibility	KEGG	Kyoto Encyclopedia of Genes and Genomes
AA	amino acid	BP	biological process
DM	dry matter	MF	molecular function
CP	crude protein	GSEA	gene set enrichment analysis
EE	ether extract	PCoA	Principal Coordinate Analysis
ADG	average daily gain	LEfSe	linear discriminant effect size
S × Q	interaction between soybean meal types and quercetin levels		
